# Machine learning approach for prediction of hearing preservation in vestibular schwannoma surgery

**DOI:** 10.1038/s41598-020-64175-1

**Published:** 2020-04-28

**Authors:** Dongchul Cha, Seung Ho Shin, Sung Huhn Kim, Jae Young Choi, In Seok Moon

**Affiliations:** Department of Otorhinolaryngology, Yonsei University College of Medicine, Seoul, South Korea

**Keywords:** Translational research, Surgical oncology

## Abstract

In vestibular schwannoma patients with functional hearing status, surgical resection while preserving the hearing is feasible. Hearing levels, tumor size, and location of the tumor have been known to be candidates of predictors. We used a machine learning approach to predict hearing outcomes in vestibular schwannoma patients who underwent hearing preservation surgery: middle cranial fossa, or retrosigmoid approach. After reviewing the medical records of 52 patients with a pathologically confirmed vestibular schwannoma, we included 50 patient’s records in the study. Hearing preservation was regarded as positive if the postoperative hearing was within serviceable hearing (50/50 rule). The categorical variable included the surgical approach, and the continuous variable covered audiometric and vestibular function tests, and the largest diameter of the tumor. Four different algorithms were lined up for comparison of accuracy: support vector machine(SVM), gradient boosting machine(GBM), deep neural network(DNN), and diffuse random forest(DRF). The average accuracy of predicting hearing preservation ranged from 62% (SVM) to 90% (DNN). The current study is the first to incorporate machine learning methodology into a prediction of successful hearing preservation surgery. Although a larger population may be needed for better generalization, this study could aid the surgeon’s decision to perform a hearing preservation approach for vestibular schwannoma surgery.

## Introduction

Vestibular schwannomas (VSs), or acoustic neuromas are benign tumors arising from the cochleovestibular nerve, which grows slowly^1^. It accounts for 6–8% of all intracranial tumors and is the most common cerebellopontine angle tumor^2^. Recently, with the help of better access to magnetic resonance imaging, the incidence has been increased, and the tumor size at the time of diagnosis has decreased^3,4^. With more early detection rates, more patients are asymptomatic when diagnosed. In these patients, the following management options may all be feasible: watchful waiting, surgery, or stereotactic radiosurgery(SRS). However, there are no clear guidelines or consensus on the optimal management of small VSs, and the optimal treatment is still under debate^5,6^. Treatment options differ individually and are dependent upon the physician’s experience, the size and growth rate of the tumor, age, patient’s preference, and hearing status. If the tumor is too big, or hearing is below serviceable hearing, hearing preservation is not essential in treatment^7^. Nevertheless, in small to medium-sized tumors with serviceable hearing, hearing preservation surgeries can be offered. Currently, middle cranial fossa approach(MCFA) and retrosigmoid approach(RSA) are the two most commonly used approaches to remove VSs.

The selection between the two approaches depends on the size and location of the tumor, and the surgeon’s preference, as each procedure has its strengths in exposing regions of the internal auditory canal or cerebellopontine angle. The preservation rate of MCFA and RSA varies among studies, ranging from 2% to as high as 93%^8^. The heterogenicity of the result makes it difficult to rate one strategy superior to another. Because hearing preservation operation takes longer and is more complex, leading to more post-surgical complications, it is reasonable to select the patients that are likely to have a decent postoperative hearing.

Recent advances in machine learning are being adopted to medical fields, especially in image recognition, including radiology, ophthalmology, histology, and dermatology^9–14^. In otology, there are relatively few, but recently, there were studies focused on the automated diagnosis of ear disease using otoendoscopy^15^, deep-learning-based noise reduction for improvement of speech recognition in cochlear implant patients^16^, and predicting the outcome of hearing in patients with sudden sensorineural hearing loss^17^. To our knowledge, despite studies focusing on predictive factors of hearing preservation surgeries^18–20^, it is hard to predict the patient’s probability of preserving auditory function following such surgery. We present a new system based on machine learning having input parameters based on preoperative data to predict the outcome of hearing preservation surgery in patients with VSs.

## Results

Fifty patients were in the cohort; 19 men and 31 women. Patient demographics are described in Table 1. The mean age at operation was 47.42 ± 11.46 years. The mean pure-tone-average(PTA) of the patients was 26.61 ± 15.64 dB HL preoperatively and 62.53 ± 41.71 dB HL postoperatively. 28 of 50 patients (56%) were able to preserve hearing following vestibular schwannoma surgery.

**Table 1:** Clinical characteristics of patients (N = 50). Preservation of hearing is classified as positive if PTA < 50 dB HL and WRS > 50% (50/50 rule).

Characteristics	Number of patients (N = 50)	
Age, mean ± SD	47.42 ± 11.46	
Sex, male:female	19:31	
Side, left:right	29:21	
Approach, MCFA:RSA	27:23	
Preservation of hearing	28(56%)	
**Influencing factors**	**Preoperative**	**Postoperative**
PTA, mean ± SD (dB HL)	26.61 ± 15.64	62.53 ± 41.71
SRT, mean ± SD (dB HL)	31.00 ± 20.05	71.04 ± 44.69
WRS, mean ± SD (%)	81.00 ± 22.90	50.08 ± 42.33
MCL, mean ± SD (dB HL)	63.50 ± 11.44	84.46 ± 26.09
I-V ABR Latency(ms)	5.62 ± 2.45	
VEMP asymmetry, mean ± SD(%)	27.45 ± 45.50	
Caloric CP, mean ± SD (%)	29.92 ± 31.64	
Tumor size, mean ± SD(mm)	13.11 ± 6.23	

PTA: Pure-tone average, SRT: Speech reception threshold, WRS: word recognition score MCL: most comfortable level, SD: standard deviation, dB HL: decibel hearing level.

mm: millimeter, ms: milliseconds.

Four machine learning models (support vector machine; SVM, gradient boosting machine; GBM, deep neural network; DNN, diffuse random forest; DRF) were compared regarding accuracy. The SVM based model showed approximately 62% percent, which is poor performance compared to the other three models. Three models (GBM, DRF, and DNN based models) exhibited a reasonable accuracy of near 90% in 5-fold-cross-validation. The comparison between the four models is summarized in Table 2. Additionally, we also explored feature importance to determine factors affecting the prediction of postoperative hearing preservation. Although feature importance tends to vary among different models, preoperative word recognition score(WRS) was the universally important feature, which was the most crucial factor in DNN and GBM models and fourth in the DRF model (Table 3).

**Table 2:** Results of four machine learning models.


Model	Accuracy	Sensitivity	Specificity	PPV	NPV	F1 score
DNN	0.90	0.93	0.86	0.90	0.90	0.91
GBM	0.88	0.86	0.91	0.92	0.83	0.89
DRF	0.86	0.89	0.82	0.86	0.86	0.88
SVM	0.62	0.92	0.23	0.60	0.71	0.73

Upper section: Confusion matrix of DNN (left) and SVM (right) model.

Predicted Y and Actual Y indicates true positive (machine predicted as hearing preservable, postoperative hearing were actually preserved).

Lower section: Detailed results of four models, sorted by accuracy and F1 score.

PPV: positive predictive value, NPV: negative predictive value.

**Table 3:** Top five feature importance among the three models.

Rank	DNN	GBM	DRF
1^st^	WRS	WRS	PTA(3 K)
2^nd^	VEMP	PTA(3 K)	PTA(8 K)
3^rd^	Tumor Size	SRT	MCL
4^th^	Caloric-CP	PTA(8 K)	WRS
5^th^	I-V Interval	I-V Interval	SRT

## Discussion

Management of VSs depends on the individual patient’s status and often relies on the experience of physicians between observation, surgery, or stereotactic radiosurgery(SRS). Since increased usage of magnetic resonance imaging led to earlier diagnosis, more patients are now asymptomatic, and watchful observation is often the choice. When it comes to SRS, the tumor control rate is comparable to conventional microsurgery^21^. It was able to maintain serviceable hearing at four years in 72.2% of the total patient in a study^22^. However, in a study that observed for a longer time, only 23% of the patient’s hearing was preserved following ten years of treatment^23^.

When it comes to surgical resection of VSs, not all hearing preservation approaches of VS microsurgery could spare hearing. The preservation rate ranges from as low as 2% to 93%^8^. Therefore, it is reasonable to select the patient preoperatively who could benefit from such hearing approaches. Previous studies established a possibility for prediction of the prognosis of hearing preservation surgery in VSs^18–20^. Also, there are studies on intraoperative findings of tumor origin, SVN(superior vestibular nerve), and IVN (inferior vestibular nerve), and concludes SVN originating tumors is associated with better hearing preservation^19,20,24^. Preoperative determination of tumor origin (SVN or IVN) has some controversies; a study by Ushio *et al*. demonstrates no significant correlation of localizing tumor origin^25^. On the other hand, other papers show the usefulness of caloric and vestibular-evoked myogenic potential(VEMP) for determining tumor origin^24,26^.

In terms of evidence, this study focuses on previous findings that exhibited the correlation of preoperative tests with hearing outcomes. Preoperative PTA of each frequency, speech reception test results, caloric test results, VEMP asymmetry, and size and location of the tumor are all put into the input parameter of the proposed system. There is also a research with an emphasis on TEOAE (transient evoked otoacoustic emissions) pattern as a prognostic factor, where patients with preserved hearing tend to have TEOAE response in all five frequency (1, 1.5, 2, 3, 4 kHz) bands^27^.

This study is, in a sense, an ensemble of several studies on predictive factors in hearing preserving VS surgery. With feature importance search, the most important factor seems to be WRS, which consistently was among the top essential features (Table 3), and it is in line with previous studies. Better preoperative WRS implies better hearing function and may indicate less vestibulocochlear nerve degeneration due to VSs. In retrocochlear lesions, WRS is usually lower than expected compared to PTA, which is commonly due to vascular compromise or toxic protein secretion by the tumor^28^, and better WRS indicates less damage to the nerve, which increases the chance of sparing nerve function in surgery.

Several efforts were made to increase the accuracy of the model. Although there are still debates on whether caloric tests can represent SVN function and VEMP tests can reflect IVN function, at least in some studies, they tend to correlate. These factors were calculated in the model and contributed to increased accuracy. In contrast, training with TEOAEs as input parameters did not improve accuracy. Instead, overall accuracy was decreased, and the gap between training loss and validation loss was increased, which indicates a more overfitting tendency in machine learning. As more input parameters are put, the system becomes vulnerable to overfitting, leading to reduced overall accuracy. In the DNN model, the number of hidden layers was 50 and 20 layers. Increasing the number of hidden layers to 200, 200, and 50 layers (wider and deeper network) led to a broader gap between training loss and validation loss, and lead to worse results, the accuracy of 0.8; again, implying overfitting.

Besides machine learning strategies, conventional methods using simple logistic regression with ROC analysis were tried to predict the outcome of VS surgery. Using WRS as a cutoff, we were able to get 82% accuracy with 92.86% sensitivity and 68.18% specificity. Using PTA(3 K) yielded 86% accuracy with 89.29% sensitivity and 81.82% specificity (optimal values were chosen using Youden’s J metrics).

Although the accuracy difference between DNN model and other classical machine learning models (SVM, GBM, linear regression) is somewhat small, there are still potential benefits of the DNN model. There are studies on the application of combining multi-omics data into individual subnetworks, then merging altogether for a prediction model^29,30^. A similar approach could be applied to this study in the future by combining the current study’s data with radiologic and genomic data subnetworks, possibly gaining more accuracy and reliability.

The design of the study is based on domain-specific knowledge. Results of the previous studies to predict hearing preservation in VS surgery were utilized for feature engineering. In the current machine learning model, feature engineering of input variables was based on previous studies on possible predictors of hearing preservation. Likewise, this study’s design could be applied to other fields of medicine, possibly yielding high accuracy of prediction.

Thanks to the nature of the prediction system based on machine learning, each patient can be individually predicted whether he/she could preserve hearing after VS surgery with an accuracy of 90 percent. However, we believe this accuracy does not mean that physicians could rely solely on algorithms, while our prediction system’s result may provide an important reference in the decision-making process. We think the treatment of VS should be based on individualized care. It requires a delicate assessment of risks and benefits when it comes to selection between watchful observation, surgical resection, and stereotactic radiosurgery. The attending physician should undergo a comprehensive review of the surgeon’s skills, patient’s preference, symptoms, tumor characteristics, and make a decision, putting it altogether. Our prediction system’s result may provide an additional factor in the decision-making process.

There are limitations to this study. The total number of patients is only 50, and the deep learning system can not reach its potential performance, and it may be prone to overfit. Overfitting may cause lower accuracy in the test set or unseen data. Although the current number is small in the field of machine learning, it is relatively big considering the rarity of VSs and even more rarity of hearing preservation surgery candidates in the medical field. In the future, if we have more data, we may reinforce the system to generalize better, and thus, predict better.

## Conclusion

This is the first study to incorporate machine learning methodology into a prediction of hearing preservation surgery. The system is built based on evidence from previous studies and could aid physicians in deciding whether to perform hearing preservation surgery on patients with VSs with a serviceable hearing status. With better patient selection using our system, individualized medical care may result in better patient outcomes.

## Materials and Methods

### Study approval

This retrospective study was approved by the Severance Hospital Institutional Review Boards (IRB number 2019-1867-001). The need for written informed consent was waived by the approval process of the review boards, owing to the retrospective nature of the study. All methods were performed complying with the Declaration of Helsinki.

### Patient selection

Among patients diagnosed with VSs from 2007 to 2017, 52 patients underwent hearing preservation surgery, either via MCFA or RSA. While all patients were included in the analysis, two patients were excluded. One patient was initially considered as VS but later revealed to be facial nerve schwannoma, which is not relevant to hearing abilities. Another patient was diagnosed as Neurofibromatosis type II. Therefore, we included a total of 50 patient’s data in the machine learning model. The detailed patient characteristics are described in Table 1.

### Data acquisition, selection, and patient classification

Electronic medical records of 50 pathologically confirmed vestibular schwannoma patients via MCFA or RSA for excision were obtained. After reviewing previous literature for possible predictors of postoperative hearing^8,18–20,24,27^, the following preoperative measures were put into the learning model: 1) Pure-tone threshold of each frequency, 2) maximal word recognition score(WRS), speech detection threshold(SDT), most comfortable level of hearing(MCL), 3) Auditory brainstem response(ABR) latency of wave I-V interval, 4) asymmetry ratio of vestibular-evoked myogenic potential (VEMP), 5) canal paresis(CP) in caloric test of affected site, 6) maximum diameter of the tumor, and 7) type of approach (RSA or MCFA). Since the model was aimed to predict postoperative hearing preservation with preoperative tests, all intraoperative factors were not taken into account. Since RSA and MCFA are preferable in VSs in the cerebellopontine angle: porus and fundus, respectively, it was treated as the relative tumor location and included in preoperative measures.

We used a binary classification for prediction modeling. The result of the patient’s postoperative hearing was classified as preserved if the patient was able to maintain the pure-tone average better than 50 decibels, and the word recognition score was above 50% (50/50 rule) at six months postoperative audiology test.

### Feature engineering, machine learning models

Some of the patient’s data were not available. VEMP asymmetry data was not available in 22 patients; CP in 1 patient; and I-V interval of ABR latency in 10 patients. In these cases, the median value was filled up for the machine learning model to minimize the missing effects. If the I-V interval of ABR latency was not countable, ten milliseconds were used. The VEMP asymmetry was calculated as the difference ratio of peak-to-peak amplitude between normal and pathologic P13 and N23 wave amplitude:

1 $$VEMPar( \% )=100\times \frac{(Ah\mbox{--}Ap)}{(Ah+Ap)},$$

where Ah is the amplitude of P13 and N23 wave on the healthy side, and Ap is on the pathologic side. All variables were classified as continuous variables, except approach type (RSA and MCFA), which was the only categorical variable in the model.

The machine learning was performed in a supervised manner. Currently, boosting and bagging are the most popular methods among tabular datasets, choosing GBM and DRF as one of the models’ lineup. SVMs are somewhat classical and were included in the study for comparison of accuracy. Lastly, neural network models have shown to be effective not only in computer visions, but also in tabular datasets, and were added to our models’ lineup. Totally, four learning models (SVM, GBM, DNN, DRF) were trained, and a comparison between the models was performed regarding accuracy. MATLAB2019a® (MathWorks, Inc., Natick, Massachusetts, United States) was used for SVM based model. For DRF, GBM, and DNN models, we built the system with Pytorch (www.pytorch.org) in Python programming language. In the training process, 80% of the patient’s data were used for training; 20% were left out for validation. We conducted five-fold-cross-validation for each model to rule out selection bias. The detailed composition of the model is described in Table 4.

**Table 4:** Detailed characteristics of three learning models.

Model		Characteristics		
		Minimum	Maximum	Mean
GBM Number of trees: 63	Depth	5	6	5.96
	Leaves	9	25	17.68
DRF Number of trees: 45	Depth	3	6	4.49
	Leaves	4	11	8.13
DNN	Structure	Input: 23, Hidden: 50 neurons (first layer), 20 neurons (second layer), Output: 2, In-place ReLU and Batch Normalization each after hidden layer		
	Learning strategy	Discriminative learning rates (Initial learning rate of 0.003 for 15 epochs, followed by rate of 0.00001 for 25 epochs) Batch size = 25, Mixed precision training of FP16 and FP32		
All models	Continuous variables	Pure-tone (250, 500, 1 K, 2 K, 3 K, 4 K, 8 K Hertz, decibels), SRT(decibels), WRS(percent), MCL(decibels), ABR I-V interval (milliseconds), Tumor size (largest diameters in millimeters) VEMP-asymmetry (percent),Caloric-CP (percent)		
	Categorical variable	Approach (RSA or MCFA)		
	Training, Validation	Training set: 80%, Validation set: 20% Five-fold cross-validation for assessment of accuracy		

GBM: gradient boosting machine, DNN: Deep neural network, DRF: diffuse random forest.

FP16: Half precision floating-point, FP32: Single-precision floating-point format.

ReLU: Rectified Linear Unit.

*The feature values were not scaled and used in as is.

## Supplementary information

Supplementary information.

## Data Availability

Patient data are not available for public access regarding patient privacy concerns but are available from the corresponding author on reasonable request if approved by the institutional review boards of Yonsei university college of medicine. DNN based machine learning model will be available with a reasonable request for testing purposes.
