# Supplementary material for: Machine learning approach for prediction of hearing preservation in vestibular schwannoma surgery
Source: Sci Rep. 2020 Apr 28;10:7136. doi: 10.1038/s41598-020-64175-1 (PMC7188896; doi:10.1038/s41598-020-64175-1)
Supplement: Supplementary file 1 — Supplementary information. [file 41598_2020_64175_MOESM1_ESM.pdf]

Machine learning approach for prediction of hearing preservation in vestibular schwannoma surgery

Dongchul Cha[a], Seung Ho Shin[a], Sung Huhn Kim[a], Jae Young Choi[a], *In Seok Moon[a]

[a]Department of Otorhinolaryngology, Yonsei University College of Medicine

Address correspondence and reprint request to:

In Seok Moon, MD, PhD

Department of Otorhinolaryngology,

Yonsei University College of Medicine

50-1 Yonsei-ro, Seodaemun–gu, Seoul, 03722, Republic of Korea

Tel: 82-2-2228-3606, Fax: 82-2-393-0850; Email: ISMOONMD@yuhs.ac

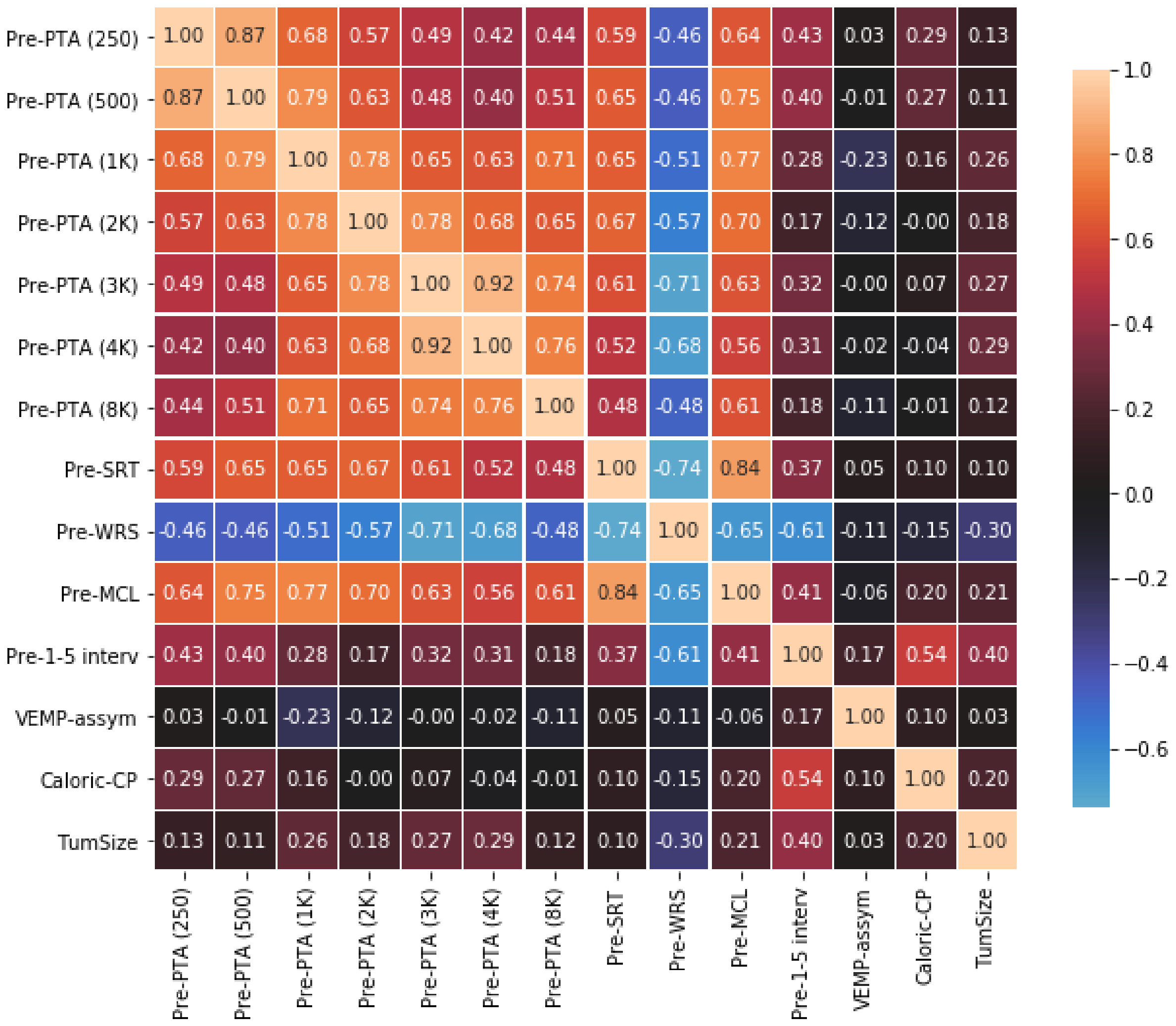

Supplementary figure 1) Correlations between features. PTAs, SRT, MCL are better hearing when lower, WRS is better hearing when higher; therefore a negative correlation is expected

Model	Accuracy	Sensitivity	Specificity	PPV	NPV	F1 score
Logistic regression (WRS)	0.82	0.93	0.68	0.79	0.89	0.85
Logistic regression (PTA-3K)	0.86	0.89	0.82	0.86	0.86	0.87
Generalized Linear model	0.86	0.86	0.86	0.89	0.83	0.87
Decision trees	0.66	0.82	0.45	0.66	0.67	0.73

PPV: positive predictive value, NPV: negative predictive value

Supplementary Table 1) Results of traditional algorithms
